# Relation of Plasma Tissue Kallikrein Levels to Presence and Severity of Coronary Artery Disease in a Chinese Population

**DOI:** 10.1371/journal.pone.0091780

**Published:** 2014-03-13

**Authors:** Qin Zhang, Xiao Ran, Dao Wen Wang

**Affiliations:** 1 Department of Anesthesia, Tongji Hospital, Tongji Medical College, Huazhong University of Science and Technology, Wuhan, China; 2 Department of Emergency, Tongji Hospital, Tongji Medical College, Huazhong University of Science and Technology, Wuhan, China; 3 The Institute of Hypertension and Department of Internal Medicine, Tongji Hospital, Tongji Medical College, Huazhong University of Science and Technology, Wuhan, China; University of Washington School of Medicine, United States of America

## Abstract

**Objectives:**

Tissue kallikrein (TK) has been shown to provide cardiovascular and cerebrovascular protective effects in animal models. The aim of this study was to investigate the relationship of plasma TK levels with the presence and severity of coronary artery disease (CAD) in the Chinese.

**Methods:**

The study involved 898 consecutive CAD patients and 905 ethnically and geographically matched controls. CAD was angiographically confirmed in all the patients, and the severity of CAD was expressed by the number of affected vessel and coronary artery stenosis scores. Plasma TK levels were measured using an enzyme-linked immunosorbent assay.

**Results:**

Plasma TK levels were significantly higher in CAD patients than controls (0.347±.082 vs. 0.256±0.087 mg/L, P<0.001), and elevated plasma TK levels were directly associated with a higher risk of CAD (OR = 3.49, 95% CI 2.90–4.19). One-way ANOVA and multivariable stepwise linear regression analysis demonstrated that TK levels were negatively associated with the severity of CAD according to vessel scores (P<0.001) and stenosis scores (r = −0.211, p<0.001).

**Conclusions:**

Our findings suggest that higher levels of TK in plasma are associated with the presence of CAD and are a predictor of mild coronary arteriosclerosis.

## Introduction

Coronary artery disease (CAD) is the leading cause of death in the western world [1], and absolute numbers of CAD events will increase dramatically in China from 2010–2029 [2]. Almost 300 variables are statistically associated with CAD [3]. Blood pressure is directly related to the risk of CAD, as well as male gender, diabetes, age, history of hyperlipidemia, family history of CAD, smoking, alcohol drinking, systolic blood pressure (SBP), diastolic blood pressure (DBP), triglyceride (TG), total cholesterol (TC), high density lipoprotein cholesterol (HDL), low density lipoproteins cholesterol (LDL), apolipoprotein A1(apo A) and B(apo B), body weight and body-mass index (BMI) [4]–[7]. To implement effective treatment and prevention strategies, the major risk factors of CAD need to be identified. In addition, these risk factors can explain only a subgroup of CAD patients [3], [8], which suggests that more risk factors need to be identified to explain the remaining patients. However, there are many endogenous protective factors against vascular injury and left ventricular remodeling, such as endothelial nitric oxide synthase, atrial natriuretic peptide and tissue kallikrein-kinin/bradykinin system [9], [10]. Their association with CAD patients remains to be elucidated.

The kallikrein-kinin system is an endogenous metabolic cascade, triggering of which results in the release of vasoactive kinins. This complex system includes the precursors of kinins known as kininogens and mainly tissue and plasma kallikreins, which liberate kinins from low - and high molecular weight kininogen. The tissue kallikreins are serine proteases encoded by highly conserved multi-gene families. Tissue kallikrein (TK), encoded by gene KLK1, cleaves kininogen to produce the potent bioactive compounds kinin and bradykinin, which have been shown to reduce elevated blood pressure and protect the heart in human and animal models [11], [12], [13]. Using transgenic and somatic gene transfer approaches to achieve a continuous supply of kallikrein–kinin in vivo, the tissue kallikrein–kinin system (KKS) exhibits protective effects in hypertension, associated insulin resistance in type 2 diabetes, cardiovascular, renal and central nervous systems via suppression of oxidative stress [13], [14]. Our previous study demonstrated that overexpression of TK attenuated type 2 diabetes-induced hypertension and renal damage [15], [16]. More recently, it was found that the pleiotropic effects of TK include inhibition of apoptosis, inflammation, proliferation, hypertrophy and fibrosis, and promotion of angiogenesis and neurogenesis in different experimental animal models [17].

Spontaneously hypertensive rats treated with TK demonstrated significant increases in the survival time after prolonged coronary artery ligation [18]. TK plays a major cardio-protective role by reducing infarct size, improving cardiac function and attenuating myocardial remodeling [19], [20]. Experiments using mice deficient in TK showed the cardio-protective effects of ischemic and pharmacological preconditioning in the myocardium. Similarly, bradykinin administration attenuated infarct size in an isolated perfused heart model of ischemia–reperfusion injury [21]. Moreover, TK gene transfer improved cardiac function, and reduced myocardial infarct size, incidence of ventricular fibrillation and apoptosis after acute ischemia–reperfusion [22]. Furthermore, TK enhances neovascularization in ischemic hearts [23]. Taken together, these data indicate a potential function of TK in protection against ischemic heart disease.

Epidemiological studies have found an inverse relationship between urinary kallikrein levels and blood pressure in patients with essential hypertension [24]. We also demonstrated that plasma TK level was negatively associated with first-ever stroke and the recurrence of stroke in a multicenter case-control study in China [25]. However, the relationship of TK and CAD is not clear. In the present study, we determined TK levels in plasma and investigated the association with the presence and severity of CAD in the Chinese Han population.

## Methods

### Study Population and Data Collection

The study protocol was approved by the institutional review board of Tongji hospital and informed written consent was obtained from all participants. The primary study population has been described previously [26]. We recruited 898 consecutive patients with angiographically documented CAD from November 2006 to November 2009 from Tongji hospital in Wuhan, China. CAD was defined as one or more of the following diagnostic criteria: (1) patients who were documented by coronary angiography to have at least a 50% stenosis in a major epicardial artery; (2) patients who survived an acute myocardial infarction and were scheduled for coronary angiography (>1 month ≤1 year from onset); and (3) patients with a history of coronary artery bypass graft or percutaneous coronary intervention. The angiograms were assessed by 2 cardiologists who were unaware that the patients were being reviewed for the study. The severity of CAD in patients was assessed and scored according to vessel scores and stenosis scores [27]. Vessel scores range from 0 to 3, depending on the number of vessels with a significant stenosis (>50% luminal stenosis). Subjects with congenital heart disease, cardiomyopathy, valvular disease, neurological diseases and renal or hepatic disease were excluded from the study. Furthermore, patients suffering from any acute illness (other acute cardiac disease, such as decompensated heart failure, decompensated valvular disease, or acute noncardiac disease, such as infection, endocrine disease or any type of surgery within the previous 3 months) were excluded as well.

905 ethnically and geographically matched controls were randomly selected from healthy residents in the community. All control subjects were free of neurological and cardiovascular diseases following the same exclusion criteria as the cases. They were also asked for a detailed medical history and were subjected to a physical examination of the cardiovascular and neurological systems and stress test.

All participants underwent a standardized interview, and data on age, sex, body weight, BMI, SBP, DBP, TG, TC, HDL, LDL, apo A, apo B, family history of CAD, hyperlipidemia, diabetes, hypertension, smoking habits and alcohol consumption were recorded. The criteria of them had been described in previous study [25], [26].

### Plasma TK Assays

Plasma samples were collected after overnight fasting and stored at −80°C. Plasma TK levels were measured using a BA-ELISA kit, which uses a double antibody sandwich enzyme immunoassay technique with Biotin-Avidin detection system and allows for the specific detection of human TK. Polyclonal antibody specific for TK was pre-coated onto microplates and incubated for 24 hours at 4°C. After aspirating each well and washing, 1% BSA-PBS was used as blocking buffer for 2 hours at 37°C. Standards and samples are then added to the appropriate microplate wells with a biotin-conjugated monoclonal antibody prepared specifically for human tissue kallikrein. Simultaneously, avidin conjugated to horseradish peroxidase is added to each microplate well and incubated. Then a TMB substrate solution is added to each well. The enzyme-substrate reaction is terminated by the addition of a sulphuric acid solution and the color is measured at 450 nm [25].

### Statistical Analysis

Demographics and clinical and laboratory variables were generally described as means ± standard deviation for continuous variables and as proportions for categorical variables. Differences between the means of the 2 continuous variables were evaluated by the independent samples t-test. Differences between noncontinuous variables were tested by χ2 analysis. Pearson’s and Spearman’s correlations were used to test the relationships between continuous variables or noncontinuous variables. One-way ANOVA and the chi-square test were used to analyze the relationships among vessel scores and general characteristic of CAD patients. Multivariable stepwise linear regression analysis was performed to determine the relationships of plasma TK levels with traditional cardiovascular risk factors and stenosis scores with general characteristic of CAD patients.

All subjects were grouped by quartiles according to plasma TK levels. Then, plasma TK levels were used as continuous variables and categorical variables in unconditional logistic regression analysis. It was performed to estimate odds ratio (OR) and 95% confidence intervals (CIs) for the association between TK in plasma and the presence of overall CAD considering the traditional risk factors of CAD described above. Statistical and association analyses were performed using SPSS 15.0 (SPSS Inc., Chicago, Illinois, USA).

## Results

### Characteristics of Study Population

A total of 898 CAD patients and 905 controls were enrolled and all subjects were available for analysis. The demographic and clinical characteristics of the CAD and control groups are shown in Table 1. As expected, CAD patients were more likely to be male (*P*<0.001). They had a higher prevalence of hypertension (*P*<0.001), diabetes (*P*<0.001), dyslipidemia (*P*<0.001), smoking (*P*<0.001) and alcohol consumption (*P*<0.001), as well as increased body weight (*P*<0.001), BMI (*P* = 0.012), older age (*P*<0.001), higher level of TC (*P*<0.001) and apo B (*P*<0.001) and lower levels of HDL cholesterol (*P*<0.001). There were no significant differences between the two groups regarding SBP, TG, LDL, apo A and family history of CAD. Notably, plasma TK levels was significantly higher in CAD patients than in controls (0.347±.082 versus 0.256±0.087 mg/L, *P*<0.001; Table 1).

**Table 1:** General characteristics of CAD patients and controls.

Variable	CAD patients	controls	P value
N (male %)	898 (78)	905 (31)	<0.001
Age, y	60±12	50±9	<0.001
Hypertension, no. (%)	386 (43)	84 (9)	<0.001
Diabetes, no. (%)	168 (19)	41 (5)	<0.001
Hyperlipidemia, no. (%)	100 (11)	32 (4)	<0.001
Alcohol consumption, no. (%)	288 (31)	145 (16)	<0.001
Current smoker, no. (%)	476 (53)	208 (23)	<0.001
Family history of CAD, no. (%)	58 (7)	76 (8)	0.117
Weight, kg	67±12	61±10	<0.001
BMI, kg/m^2^	24±5	23±3	0.012
SBP, mm Hg	132±21	123±19	0.862
DBP, mm Hg	80±14	77±11	<0.001
Total cholesterol, mmol/L	4.24±1.08	4.09±0.97	<0.001
Triglyceride, mmol/L	1.86±1.61	1.12±0.76	0.802
HDL, mmol/L	1.04±0.29	1.48±0.34	<0.001
LDL, mmol/L	2.43±0.84	2.62±0.80	0.493
Apo A, g/L	1.14±0.39	1.12±0.33	0.321
Apo B, g/L	0.78±0.31	0.88±0.27	<0.001
TK levels, mg/L	0.347±0.082	0.256±0.087	<0.001

Abbreviations: BMI, body mass index; SBP, systolic blood pressure; DBP, diastolic blood pressure; HDL, high-density lipoprotein cholesterol; LDL, low-density lipoprotein cholesterol; Apo A, apolipoprotein A; Apo B, apolipoprotein B; TK, tissue kallikrein.

### Association of TK Levels with CAD

Univariable correlations between TK and atherogenic risk factors such as age, sex, body weight, BMI, SBP, DBP, TG, TC, HDL, LDL, apo A, apo B, family history of CAD, history of hyperlipidemia, diabetes and hypertension in controls were showed in Table 2. There was no statistical correlation among them in univariable correlations and multivariable stepwise linear regression analysis, except for hypertension (Bata = −3.71; P<0.001), TC (Bata = 3.78; P<0.001); HDL (Bata = 2.29; P = 0.022) and Apo A (Bata = 2.29; P = 0.023).

**Table 2:** The relationship between TK levels and cardiovascular disease risk factors in controls subjects.

Variable	Quarter 1	Quarter 2	Quarter 3	Quarter 4	P value
TK levels, mg/L	<0.236	0.236–0.293	0.293–0.357	>0.357	
No. of participants	396	280	128	101	
Male sex, no. (%)	129(33)	95(34)	31(24)	29(29)	0.172
Age, y	50±9	48±9	51±10	52±8	0.213
Hypertension, no. (%)	57(14)	15(5)	5(4)	7(7)	<0.001
Diabetes, no. (%)	20(5)	10(4)	4(3)	7(7)	0.876
Hyperlipidemia, no. (%)	23(6)	7(3)	3(2)	1(1)	0.016
Alcohol consumption, no. (%)	65(16)	49(18)	19(15)	12(12)	0.298
Current smoker, no. (%)	101(26)	62(22)	23(18)	22(22)	0.153
Family history of CAD, no. (%)	39(10)	21(8)	10(8)	6(6)	0.175
Weight, kg	61±9	61±10	59±10	60±8	0.733
BMI, kg/m^2^	23±3	23±3	23±3	23±3	0.707
SBP, mm Hg	124±19	122±18	124±17	129±18	0.857
DBP, mm Hg	77±12	76±11	78±10	79±10	0.612
Total cholesterol, mmol/L	4.59±0.89	4.59±0.79	4.56±0.99	4.53±0.89	0.271
Triglyceride, mmol/L	1.13±0.65	1.09±0.62	1.14±0.97	1.16±1.13	0.134
HDL, mmol/L	1.47±0.32	1.49±0.36	1.55±0.34	1.51±0.36	0.375
LDL, mmol/L	2.62±0.79	2.66±0.63	2.54±0.77	2.61±0.67	0.547
Apo A, g/L	1.13±0.34	1.09±0.33	1.13±0.34	1.19±0.31	0.296
Apo B, g/L	0.76±0.28	0.79±0.27	0.77±0.46	0.86±0.35	0.259

Abbreviations: TK, tissue kallikrein; BMI, body mass index; SBP, systolic blood pressure; DBP, diastolic blood pressure; HDL, high-density lipoprotein cholesterol; LDL, low-density lipoprotein cholesterol; Apo A, apolipoprotein A; Apo B, apolipoprotein B. All the subjects (898 CAD patients and 905 controls) were grouped by quarters according to plasma TK levels: Quarter 1 (396 controls and 54 cases), Quarter 2 (280 controls and 171 cases), Quarter 3 (128 controls and 323 cases), and Quarter 4 (101 controls and 350 cases). Data are means ±SD. P-values for the difference among the 4 groups, tested with One-way ANOVA and Chi square test.

We grouped all the subjects by quarters and found that the group with higher plasma levels of TK had more CAD patients. Logistic regression analysis considering traditional risk factors showed a positive correlation between plasma TK levels and the presence of CAD when plasma TK levels were used as a continuous variable (OR = 3.49; 95% CI, 2.90 to 4.19). As compared with the first quarter, the odd ratios for CAD were as follows: second quarter, 7.17 (95% CI, 4.34 to 11.83); third quarter, 41.27 (95% CI, 24.22 to 70.33); fourth quarter, 48.08 (95% CI, 28.15 to 82.13). These outcomes indicate that plasma TK level was independently, in a dose–response manner, associated with increased risk of CAD (Table 3).

**Table 3:** Unconditional logistic regression analysis for CAD and TK levels.

Groups measure	Quarter 1	Quarter 2	Quarter 3	Quarter 4
TK levels, mg/L	<0.236	0.236–0.293	0.293–0.357	>0.357
Total CAD, NO.	54	171	323	350
Unadjusted (ORs)†	1	4.48 (3.18–6.31)	18.51(13.04–26.27)	25.41(17.72–36.44)
*P*		<0.001	<0.001	<0.001
Adjusted (ORs)†	1	7.17 (4.34–11.83)	41.27(24.22–70.33)	48.08(28.15–82.13)
*P*		<0.001	<0.001	<0.001

Abbreviations: TK, tissue kallikrein; ORs, odds ratios.

† ORs (95% confidence interval). Adjusted ORs have been adjusted for age, sex, presence or absence of hypertension, diabetes, hyperlipidemia, alcohol consumption and smoking, family history of CAD, weight, BMI, SBP, DBP, total cholesterol, triglyceride, HDL, LDL, Apo A and Apo B.

Differences in Plasma TK levels among CAD patients with and without traditional cardiovascular risk factors were not significant, except for apo B (r = 0.077, P = 0.034), current smoking history (r = −0.081, P = 0.015) and male sex (r = −0.091, P = 0.007). A stepwise multiple linear regression analysis showed that TG (Bata = 2.51; P = 0.012) and smoking habits (Bata = −2.59; P = 0.010) enter the regression equation in CAD.

### TK Levels and CAD Severity

Patients with CAD were further subclassified into 4 subgroups (Group 0, Group 1, Group 2, and Group 3) according to the number of significantly affected vessels. Plasma TK levels and traditional cardiovascular risk factors in different groups were presented in Table 4. It is interesting that plasma TK levels were apparently decreased with increasing vessel scores (P<0.001; Table 4).

**Table 4:** The relationships between vessel scores and general characteristic of CAD patients.

Variable	Group 0	Group 1	Group 2	Group 3	P values
N (male %)	38 (79)	181 (79)	234 (77)	445 (78)	0.96
Age, y	63±11	57±11	59±11	61±10	<0.001
Hypertension, no. (%)	18(47)	70(39)	91(39)	207(47)	0.136
Diabetes, no. (%)	7(18)	14(8)	62(26)	85(19)	<0.001
Hyperlipidemia, no. (%)	1(3)	14(8)	38(16)	47(11)	0.011
Alcohol consumption, no. (%)	12(32)	65(36)	83(35)	128(29)	0.195
Current smoker, no. (%)	16(42)	99(55)	119(51)	242(54)	0.425
Family history of CAD, no. (%)	5(13)	8(4)	14(6)	31(7)	0.226
Weight, kg	67±9	68±11	68±10	67±10	0.706
BMI, kg/m^2^	24±3	24±3	24±3	24±3	0.995
SBP, mm Hg	132±19	132±19	134±21	133±20	0.834
DBP, mm Hg	83±14	81±11	81±13	80±14	0.432
Total cholesterol, mmol/L	3.98±0.79	4.14±0.96	4.42±1.08	4.41±1.27	0.008
Triglyceride, mmol/L	1.66±1.27	1.56±0.95	1.91±1.25	2.01±1.94	0.012
HDL, mmol/L	1.02±0.28	1.04±0.27	1.04±0.31	1.04±0.32	0.962
LDL, mmol/L	2.37±0.53	2.34±0.83	2.44±0.81	2.45±0.94	0.538
Apo A, g/L	1.09±0.34	1.07±0.28	1.19±0.45	1.15±0.39	0.030
Apo B, g/L	0.91±0.24	0.82±0.23	0.92±0.26	0.88±0.28	0.101
TK levels, mg/L	0.384±0.063	0.359±0.087	0.353±0.077	0.335±0.081	<0.001

Abbreviations: BMI, body mass index; SBP, systolic blood pressure; DBP, diastolic blood pressure; HDL, high-density lipoprotein cholesterol; LDL, low-density lipoprotein cholesterol; Apo A, apolipoprotein A; Apo B, apolipoprotein B; TK, tissue kallikrein. CAD patients were subclassified into 4 subgroups (Group 0, Group 1, Group 2, and Group 3) according to the number of significantly affected vessels. P-values for the difference among the 4 groups, tested with One-way ANOVA and Chi square test.

On bivariate correlation analyses, stenosis scores were inversely correlated to plasma TK levels (r = −0.211; p<0.001) and directly correlated with age (r = 0.111; p = 0.001), TC (r = 0.078; p = 0.021) and TG (r = 0.100; p = 0.003). In multivariable stepwise linear regression analysis the following, listed in descending order, were found to be associated with stenosis scores: plasma TK level (Bata = −0.197; P<0.001), drinking (Bata = −0.163; P<0.001), DBP (Bata = −0.139; P = 0.003), TG (Bata = 0.127; P = 0.007), age (Bata = 0.126; P = 0.007). These data suggested that plasma TK levels were negatively associated with the severity of CAD as assessed both by the number of affected vessels and stenosis scores.

## Discussion

We examine the relationship between plasma TK levels and the presence of CAD in the Chinese Han population. The results of our study showed that CAD patients had higher plasma TK levels compared to control subjects. Logistic regression analysis indicated that this association was dose-dependent and independent of other factors related to risk for arterial disease. These data suggest that an elevated plasma TK level might be a strong and independent endogenous risk factor of CAD in the Chinese population. However, TK is known to be a cardioprotective agent [28], which exhibits a wide spectrum of beneficial effects by inhibiting of neointima formation in blood vessels, promoting angiogenesis, reducing cardiac and vascular injuries, attenuating cardiac infarction and cardiac remodeling and improving cardiac function without hemodynamic effects. It is a paradox that plasma TK level was independently and positively associated with the presence of human CAD in our study, although numerous studies have confirmed the independent cardioprotective effect of TK in animal models [18]–[22], [29].

The mechanism of increased plasma TK levels in CAD patients is uncertain. TK processes low-molecular weight kininogen substrates to releases vasoactive kinin peptides. Then, intact kinins bind to bradykinin B2 receptors activating signaling pathways such as NO-cyclic 3′,5′-guanosine monophosphate and prostacyclin–cyclic adenosine monophosphate, which trigger a broad spectrum of biological effects including vasodilatation, smooth muscle contraction and relaxation, inhibition of apoptosis, atherosclerosis, inflammation, hypertrophy and fibrosis, protection against ischemia/reperfusion damage and promotion of angiogenesis and neurogenesis [13].

The association of elevated plasma TK levels and increased presence of CAD might be explained by the following reasons. Firstly, CAD patients with a history of diabetes might have higher plasma levels of TK. Coronary artery disease is a frequent accompaniment of type 2 diabetes [30], as seen in our study as well. Campbell et al. found that TK levels are increased in type 2 diabetes [31]. Therefore, CAD patients might have elevated plasma TK levels because of a higher prevalence of diabetes. However, there was no difference in plasma TK levels between CAD patients with and without a history of diabetes in our finding. Plasma TK levels are still positively associated with the presence of CAD adjusting traditional cardiovascular disease risk factors such as history of diabetes. As a result, CAD patients have higher plasma levels of TK independent of a history of diabetes. Secondly, traditional risk factors of CAD might be impact on plasma TK levels. We, therefore, evaluated the associations between them in controls. There was no statistical correlation among them in univariable correlations and multivariable stepwise linear regression analysis, except for hypertension and lipid metabolism, Logistic regression analysis considering traditional risk factors also showed that plasma TK level was independently, in a dose–response manner, associated with increased presence of CAD. Thirdly, the effects of medication on plasma TK levels might exist. Various physiological mechanisms of the relationship between TK and KKS had been well elucidated [28]. Angiotensin-converting enzyme inhibitor or angiotensin II receptor blocker may affect the levels of plasma TK. However, a previous study found no differences in circulating levels of bradykinin and kallidin peptides, and low molecular weight kininogens, or in plasma kallikrein or kallistatin although plasma TK levels were higher in subjects with type 2 diabetes. Similarly, statin therapy did not change any variables of the circulating KKS. Aspirin, calcium antagonists, beta blockers, or long-acting nitrate therapies did not influence any KKS variables [31]. Our previous finding also demonstrated that the associations between of antihypertensive agents, insulin, and hypoglycemic drugs with plasma TK levels were not apparent. The only exception was a slight increase in stroke patients taking aspirin, which was still lower than normal controls [25]. Based on the literature and previous research, the influence of medication on plasma TK level is not apparent. Fourthly, increases in TK to compensate for a dysfunctional state in CAD patients might be the reason. It is known that patients with heart failure have elevated plasma atrial natriuretic peptide (ANP) concentrations. At the initial stage of heart failure, ANP might serve to restrain sympathetic nervous system outflow to the kidney or skeletal muscle and slow disease progression [32], [33]. It is similar with another study that TK levels were higher in subjects with type 2 diabetes [31]. TK exerts protective effects in the presence of diabetes by activating phosphatidylinositol 3-kinase/protein kinase B and adenosine 5′-monophosphate-activated protein kinase signaling pathways [15]. We hypothesize that plasma TK levels increased to provide protection against atherosclerosis. Therefore, elevated plasma TK might be a biomarker for a dysfunctional state rather than a risk factor of CAD and the mechanism remains to be elucidated in the future.

We reported the relationship between plasma TK levels and severity of CAD in the present study. The severity of CAD was evaluated using vessel scores and stenosis scores. It is interesting to note that plasma TK levels were negatively associated with the severity of CAD according to vessel and stenosis scores. These results suggest that decreased plasma TK levels might predict the severity of CAD. Numerous studies have demonstrated that TK inhibits the proliferation of cultured vascular smooth muscle cells and neointimal formation in blood vessels after balloon angioplasty stimulate endothelial cell proliferation, attenuate vascular injury by promoting vascular regeneration and accelerating spontaneous angiogenesis [13], [23], [28], [34]. Taken together, these indicate that TK may have significant implications for protecting against atherosclerosis and ischemic vascular disease and may be a prognostic tool for evaluating the extent of obstructive CAD.

Overexpression of the human TK gene in spontaneously hypertensive rats induced hypotension [35], and epidemiological studies [24], which are consistent with our findings regarding the negative association between plasma TK levels and a history of hypertension in controls. Our finding showed that plasma TK levels had an inverse correlation with a history of hyperlipidemia as well. These data suggest that plasma TK may influence blood pressure and lipid metabolism. Further prospective and basic studies are still needed to elucidate the role of TK on lipid metabolism.

In addition, our previous study demonstrated that plasma TK level was negatively associated with the risk of first-ever stroke and stroke recurrence [25]. As plasma TK levels were increased in diabetes [31] and CAD, decreased plasma TK levels might be specific biomarker in stroke.

Our study has several limitations. First, the case-control design limited our ability to establish a causal link between elevated plasma TK levels and CAD. As a result, the relationship between plasma TK level and the risk of CAD should be verified in future prospective studies. Second, even though the multivariate analysis adjusted for the traditional risk factors, it is still possible that the confounding elements cannot be “adjusted out”. Third, it was impossible to avoid the influence of medications on plasma TK levels completely, although the associations of drugs with plasma TK levels were not apparent in previous studies. Fourth, the relationship between plasma TK level and timing for the progression of cardiovascular disease might be an important evidence to indicate that TK plays an important role associated with CAD. These questions can only be addressed through additional prospective studies of the association of TK with first-ever CAD and the extent of obstructive CAD.

In conclusion, our research, combined with previous basic studies, suggest that elevated TK is positively associated with the presence of CAD and negatively associated with the severity of CAD and that they might be a strong and independent biomarker of CAD in the Chinese population. Moreover, levels of plasma TK could be a useful prognostic tool for the evaluation of the severity of CAD. Large prospective human studies, as well as cellular and animal research, are needed to elucidate why CAD patients have higher plasma TK levels.
